# An In Vitro Pilot Study Comparing the Novel HemoClear Gravity-Driven Microfiltration Cell Salvage System with the Conventional Centrifugal XTRA™ Autotransfusion Device

**DOI:** 10.1155/2020/9584186

**Published:** 2020-09-08

**Authors:** Anneloes Hoetink, Sabine F. Scherphof, Frederik J. Mooi, Paul Westers, Jack van Dijk, Sjef J. van de Leur, Arno P. Nierich

**Affiliations:** ^1^Department of Anesthesiology and Intensive Care, Isala, Zwolle, Netherlands; ^2^Division of Anesthesiology Intensive Care and Emergency Medicine, University Medical Center Utrecht, Utrecht, Netherlands; ^3^ECCare, Zwolle, Netherlands; ^4^Department of Epidemiology, UMC Utrecht Julius Center, Utrecht, Netherlands; ^5^Department of Clinical Chemistry, Isala, Zwolle, Netherlands

## Abstract

**Background:**

In 2013, the World Health Organization reported a shortage of 17 million red blood cell units, a number that remains growing. Acts to relieve this shortage have primarily focused on allogeneic blood collection. Nevertheless, autologous transfusion can partially alleviate the current pressure and dependence on blood banking systems. To achieve this, current gold standard autotransfusion devices should be complemented with widely available, cost-efficient, and time-efficient devices. The novel HemoClear cell salvage device (HemoClear BV, Zwolle, Netherlands), a gravity-driven microfilter, potentially is widely employable. We evaluated its performance in the cardiac postoperative setting compared to the centrifugal XTRA™ autotransfusion device.

**Methods:**

In a split-unit study (*n* = 18), shed blood collected 18 hours after cardiothoracic surgery was divided into two equal volumes. One-half was processed by the XTRA™ device and the other with the HemoClear blood separation system. In this paired set-up, equal washing volumes were used for both methods. Washing effectivity and cellular recovery were determined by measuring of complete blood count, free hemoglobin, complement C3, complement C4, and D-dimer in both concentrate as filtrate. Also, processing times and volumes were evaluated.

**Results:**

The HemoClear and XTRA™ devices showed equal effectiveness in concentrating erythrocytes and leucocytes. Both methods reduced complement C3, complement C4, and D-dimer by ≥90%. The centrifugal device reduced solutes more significantly by up to 99%. Free hemoglobin load was reduced to 12.9% and 15.5% by the XTRA™ and HemoClear, respectively.

**Conclusion:**

The HemoClear device effectively produced washed concentrated red blood cells comparably to the conventional centrifugal XTRA™ autotransfusion device. Although the centrifugal XTRA™ device achieved a significantly higher reduction in contaminants, the HemoClear device achieved acceptable blood quality and seems promising in settings where gold standard cell savers are unaffordable or unpractical.

## 1. Introduction

Transfusion of red blood cells (RBCs) represents one of the most frequently performed medical procedures in hospitalized patients [1]. For 2013, the World Health Organization (WHO) reported a total number of 75 million RBC units transfused worldwide.

Nevertheless, the same year was also characterized by a shortage of 17 million RBC units [2]. Allogeneic blood collection as the cornerstone of safe and sufficient blood supply is beyond dispute. However, autologous transfusion also plays a role in tackling blood shortages and partially relieves the current pressure and dependence on blood banking systems [3, 4]. Moreover, autologous blood transfusion is widely accepted as the preferred option where possible due to numerous advantages over allogeneic blood [3, 5–7]. However, limited by the availability and affordability of current cell salvage devices, autologous transfusion is mostly confined to fully equipped hospital operating rooms [4, 8, 9]. Due to the high capital investment, extensive training requirements, and expensive consumables, cell salvage is merely cost-effective in settings where hemorrhage is anticipated [10].

Additionally, the need for electricity and ponderous design makes these devices unpractical in low-resource settings [8, 11, 12]. HemoClear (HemoClear BV, Zwolle, Netherlands) presents a novel simplistic gravity-driven device for washing shed blood and concentrating RBCs, with the potential to counteract global blood shortage. This is reported here in an in vitro feasibility study of the HemoClear for washing perioperative shed blood after cardiac surgery.

Cardiac surgery is characterized by high volumes of blood loss and accounts for 15–20% of all perioperative transfusion [13]. Since 2011, the routine use of autologous transfusion in cardiac surgery setting has been recommended in multiple guidelines [14, 15]. Since shed blood typically contains high levels of complement, endotoxin, tissue factor, free hemoglobin, lipid particles, thrombo-emboli, fibrinolytics, and inflammatory cytokines, direct reinfusion of unwashed RBCs should be avoided. Besides, it is considered as potentially harmful in the current guidelines [14]. To this end, cardiac surgery rooms are often equipped with gold standard autotransfusion devices such as the XTRA™ cell salvage system (LivaNova NV, Amsterdam, Netherlands). Although these devices time-efficiently wash high blood volumes, they are also costly and often not available postoperatively or employable for washing of low volumes (<800 mL) [16–18]. Also, there are concerns about the influence of the centrifugal forces on RBC viability and quality [8].

**Figure 1:**
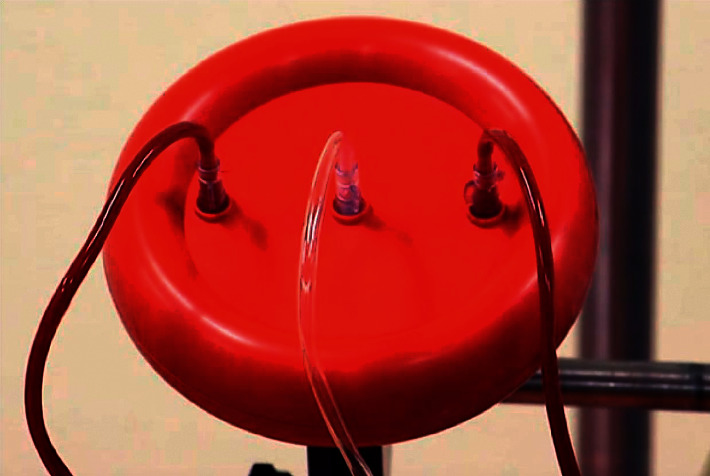
The HemoClear device at work. Shed blood enters the filter from the left inlet and is separated into concentrated RBCs that leave the filter from the right outlet and plasma that flows away in the middle outlet.

**Figure 2:**
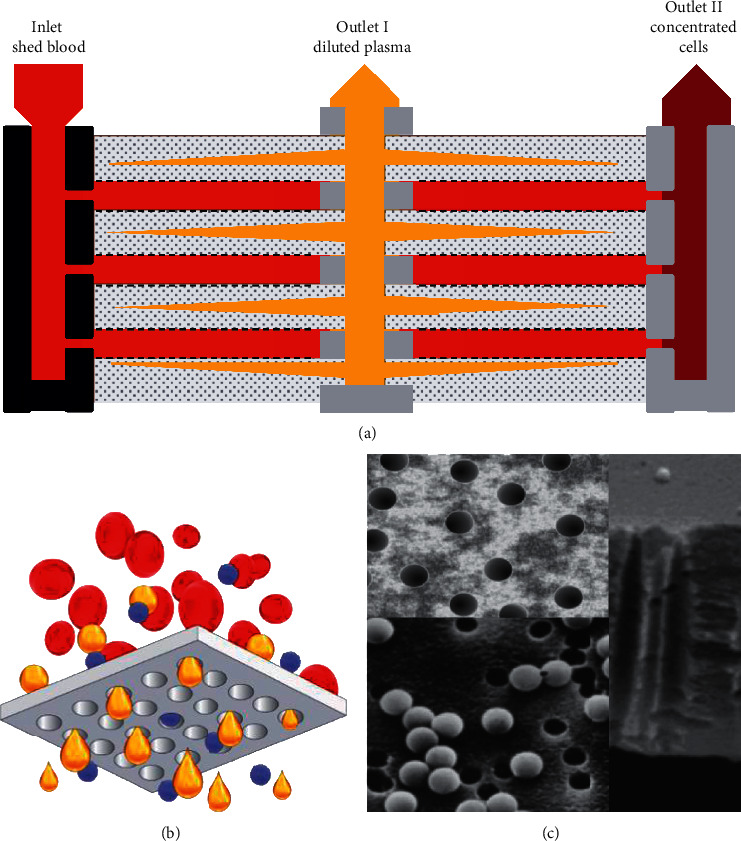
(a) Schematic illustration of the HemoClear multilayer, micropore filtration technology. The filtration module comprises an inlet for the shed blood to enter. Two outlets lead the diluted plasma (outlet I) and concentrated cells (outlet II) to collection bags. The internal design of the membrane yields a cross-flow current of shed blood cells from the inlet to outlet II, preventing blockage of the pores. (b) The pores only allow passage of fluids (yellow) and solutes (blue), while red blood cells remain on top of the filtration layer (grey). (c) Highly accurate nanotechnology is used to achieve pores of exactly 2.3 microns in diameter.

HemoClear is a disposable gravity-driven microfilter (2.3 micrometers) capable of filtering any fluid that contains red blood cells (Figure 1; Supplemental Video 1; Figure 2). To assess its performance in washing and concentrating of RBCs, the HemoClear device is compared to a routinely used centrifugal autotransfusion device, the XTRA™. Both devices were assessed based on their ability to recover the red blood cells and remove noncellular components, including free hemoglobin, fibrinolysis product D-dimer, complement component C3, and complement component C4. We hypothesize that the HemoClear cellular recovery and washing performance are comparable to those of the gold standard centrifugal device.

## 2. Methods

This in-vitro pilot study was approved by Isala IRB/Research Ethics Committee Research and Development Committee (local registration number: 180306; principle investigator: Nierich; date of registration: 6 March 2018), and written informed participation consent was obtained from each patient in order to use postoperative shed blood. This manuscript adheres to the applicable TREND guidelines [19, 20].

### 2.1. Sample Collection

The shed blood was collected from 20 patients that underwent elective coronary artery bypass grafting or valve surgery at the Isala Clinics, Zwolle, Netherlands. The sample size was not based on statistical power analysis. Patients were included based on a minimum postoperative wound fluid volume of 600 ml. Two patients were excluded from data analysis due to technical failures that arose after the XTRA™ device minimal processing volume requirement was not met. The comparative data analysis of the residual 18 patients is reported.

**Figure 3:**
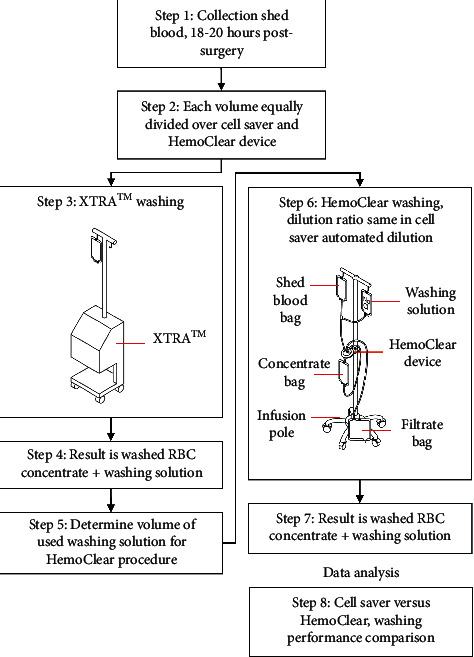
Schematic illustration of the study design. Each shed blood sample is aliquoted in two volumes, to be washed by the cell saver machine and HemoClear device. Washing with the cell saver device is performed firstly, and the automatically used washing volume determined. Subsequently, the same volume is used in the HemoClear device set-up; the HemoClear device is attached to an infusion pole. Preprocessed blood bags and processed bags are hung above and below the device, respectively.

The complete volume of shed blood was collected from each patient's drain reservoir 18 hours after cardiothoracic surgery to simulate a worst-case scenario in order to increase the number of solutes to be washed out. A prewashing sample was taken prior to equally dividing the volume into two parts, to be washed with either the XTRA™ Autotransfusion System with a 225 ml centrifugal bowl or the HemoClear device. The shed blood volume was transferred into the collection reservoir of the XTRA™ in order to retain clots before processing by both devices. Half of the volume was transferred into the first blood bag of the HemoClear device set-up in accordance with the paired set-up of the study. (Figure 3 illustrates the schematic set-up of the washing study design.)

### 2.2. Washing Procedure

Operators were limited to those who have been properly trained to use the autotransfusion devices. The same XTRA™ device was used for each case throughout the study. The XTRA™ autotransfusion machine, as the disposable HemoClear device, was set up according to manufacturers' specifications.

### 2.3. The XTRA™ Autotransfusion System

Washing procedures with the XTRA™ were performed firstly to enable calculation of the used volume of washing saline (ml NaCl 0.9%). The XTRA™ Autotransfusion System uses a cylindrical bowl of 225 ml with two indentations in the sides to increase the mixing of cells. First, it fills with 135 ml of shed blood from the prefilter 150 Mu collection reservoir. The centrifuge then operates at a speed of 10,000 rpm. Initially, the bowl fills at 600 mL/min, slowing down to 250 mL/min during the secondary bowl fill. During each wash cycle, wash fluid is pulsed through the bowl, which increases turbulence and washing efficiency. The wash volume used on average is 250 mL during each cycle.

### 2.4. The HemoClear Device

**Figure 4:**
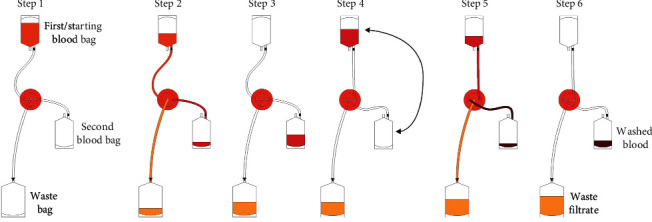
Schematic representation of the HemoClear filtration process. Step 1–3 represent first filtration round; steps 4–6 represent second filtration round.

In the filtration set-up, the HemoClear device is centrally integrated into a two-blood bag system and one waste collection bag (Figure 4, step 1). The second half of the collected shed blood into the XTRA™ reservoir was transferred into the first blood bag of HemoClear. As the initial step in each HemoClear washing round, the shed blood volume was diluted with 0.9% saline before running through the filter. In line with the paired set-up, the HemoClear overall dilution factor for each sample was equal to the dilution factor automatically applied to the matched sample during XTRA™ washing. XTRA™ dilution was calculated based on measurements of the preprocessing and postprocessing volumes of the starting volume, concentrated washed RBCs, and filtrate volume.

Directly after dilution, shed blood was processed by the HemoClear (Figure 4, steps 2-3). Filtration was driven by a gravitational force of one meter of height difference between the starting blood bag and the device. (This height difference was determined to be optimal in previous validation studies during the technical development period.) The primary goal was to reach the same concentrate end volume of the XTRA™. To reach this same volume, the concentrate was filtrated again by lifting it from its collection point at the device level to one meter above. To follow up with an additional filter round, the high-hanging empty prefiltration blood bag and the low-hanging concentrate-containing blood bag were exchanged. The first starting blood bag was lowered to the device level since it was then used as the concentrate collection bag (Figure 4, steps 4–6). Due to the filter and set-up design, no other handlings were needed and the filtration process was maintained as a closed-loop system, preventing bacterial contamination. Based upon the measured concentrate volume of the cell saver, the shed blood was passed through the filter during multiple consecutive filtration rounds. The filtrate was collected one-meter level below the device in the waste bag, which was replaced only when full.

### 2.5. Washing Quality Measurements

Washing effectivity and efficiency were determined by measuring various parameters in the preprocessed wound fluid, filtrate, and concentrated washed red blood cells, for both the HemoClear filtration system and the XTRA™ centrifugation-based procedure.

### 2.6. Laboratory Analysis

The washing performance of either device was assessed in concentrating erythrocytes and in removing other substances. Samples of the resulting RBC suspension were taken; 3 mL samples were placed in EDTA vacuum containers and labeled, and 1 ml samples in a heparinized blood gas syringe. Both the RBC suspensions as the filtrate were analyzed for platelets, white blood cells (WBC), total red blood cells, and hematocrit (HCT) using a coulter counter. The hematocrit and the hemoglobin concentration of the RBC suspension in the EDTA containers were measured using a Pentra 120 blood analyzer (ABX Horiba Diagnostics, Lier, Belgium) and a Beckmann DU-640 spectrophotometer (Beckmann Coulter, Brea, CA, USA), respectively. D-dimer, complement C3, and complement C4 levels were determined by spectrophotometer. The suspension fluid in the samples was isolated by centrifugation using a Heraeus Labofuge 400R Centrifuge (Heraeus, Hanau, Germany), and the hemoglobin concentration of this supernatant was measured using the same spectrophotometer. Based on free and bound hemoglobin levels and hematocrit, the percentage of hemolysis was calculated as follows: hemolysis (%) = (free Hb × (100- HCT))/(total Hb) × 100. Hemolysis calculations that returned values higher than 100% were corrected to 100%.

### 2.7. Processing Time

The processing time for all devices was also recorded. Times were measured from the start of processing to the production of the final product (i.e., the start of filling to the end of the empty cycle).

### 2.8. Calculation of Blood Cell Mass-Processing Rate

The starting and ending hematocrit and liquid volumes in the collection reservoir and holding bag were recorded, and the RBC recovery was calculated as follows: recovery of erythrocytes was quantified according to the following formula: RBC recovery (%) =1 ((erythrocyte concentration postprocessing filtrate × volume postprocessing)/(erythrocyte concentration preprocessing × volume preprocessing) × 100%).

### 2.9. Statistical Analysis

Results values are expressed in terms of mean ± standard deviation. Statistical significance (*P* value <0.05) of the differences compared to the baseline measurement and between the two processing devices was determined using paired two-tailed Student's *t*-tests performed in SPSS Statistics (IBM) for Windows, Version 22.0.

## 3. Results

**Figure 5:**
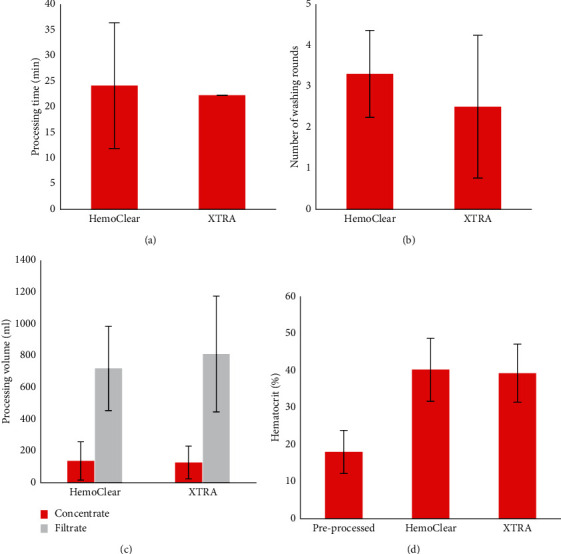
Operational assets (a, b), processing characteristics (c, d).

A total of 18 patients' postoperatively shed blood was processed using the HemoClear device and conventional cell saver. The HemoClear filtration device produced concentrated red blood cells within a timeframe (24.6 ± 12.4 min) comparable to the XTRA™ processing time (22 min ± 0 min). The mean HemoClear washing rounds (3.07 ± 0.64) were equivalent to needed XTRA™ runs (2.17 ± 0.79). The HemoClear and XTRA™ device yielded comparable concentrate volumes of 138 ± 123 mL and 125 ± 105 mL, respectively (Figure 5).

Table 1 provides an overview for comparison of the HemoClear device to the conventional cell saver.

The HemoClear filter-produced concentrates show hematocrit (from 17.9 ± 5.8 to 40.0 ± 8.4%, *P* < 0.001) and hemoglobin (from 3.71 ± 1.1 to 8.02 ± 1.7 mmol/L, *P* < 0.001) values more than doubled compared to the preprocessed samples. HemoClear on average recovered 94.8 ± 4.0% of red cells. This performance was similar to the cell saver's recovery (96.5 ± 4.5%, *P*=0.147). Overall, washing by means of either of the two devices substantially reduced the total load of free hemoglobin by about 10-fold and lowered hemolysis by approximately 5-fold (free hemoglobin and hemolysis compared to baseline *P* < 0.001, for HemoClear and the XTRA™). Hemolysis (%) rate in the HemoClear concentrate was 1.00 ± 0.76% and in the XTRA™ concentrate 0.73 ± 0.30%.

**Figure 6:**
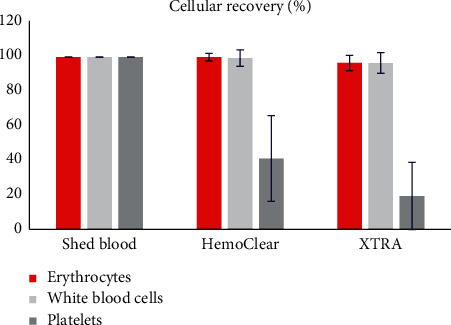
Cellular recovery in concentrated part.

Centrifugation-free washing resulted in a thrombocyte concentration of 30.8 ± 21.9 10^10^/L, while centrifugation-based washing yielded a significantly lower concentration of 16.2 ± 19.0 10^10^/L (*P*=0.003) thrombocytes. Both HemoClear (from 6.3 ± 2.8 to 15.2 ± 6.5 10^9^/L, *P* < 0.001) and XTRA™ (from 6.3 ± 2.8 to 12.8 ± 4.8 10^9^/L, *P* < 0.001) concentrated leucocytes (Figure 6).

**Figure 7:**
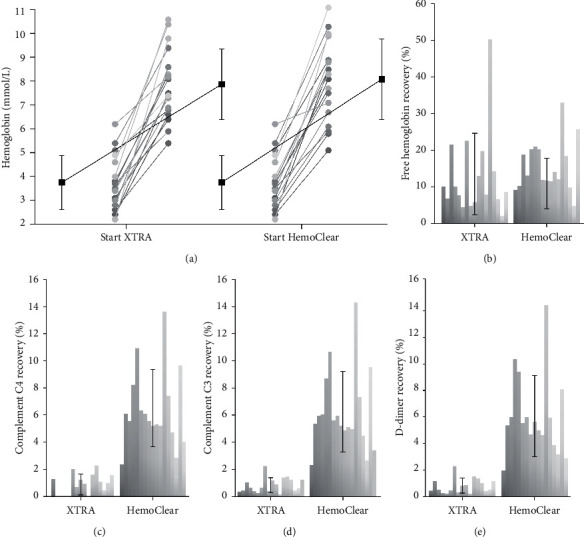
(a) Hemoglobin concentration in concentrates of XTRA™ cell saver and HemoClear before and after washing. Black lines indicate mean values, error bars represent standard deviation. (b–e) Percentage of total load recovered in concentrates of the XTRA™ and HemoClear device. Each bar represents an individual sample, error bars indicate standard deviation. (b) Free hemoglobin recovery. (c) Complement C4 recovery. (d) Complement C3 recovery. (e) D-dimer recovery.

The ability to wash out proteins, such as central complement system activators complement component C3 and complement component C4 and fibrinolysis product D-dimer, was also investigated (Figure 7). Total loads of complement C3 (6.2 ± 3.0% remaining, *P* < 0.001 compared to baseline), complement C4 (6.4 ± 2.9% remaining, *P* < 0.001 compared to baseline), and D-dimer (5.9 ± 3.0% remaining, *P* < 0.001 compared to baseline) were reduced by over 15-fold after the HemoClear washing procedure. The XTRA™ reduced these proteins more significantly to a percentage less than 1.0% remaining. Free hemoglobin was equally washed out by the two devices to a mean level of less than 15% of the total baseline load.

## 4. Discussion

This study was set out to assess the blood washing performance of the novel HemoClear device by comparison to the centrifugal autotransfusion device XTRA™ as cell saver. Postoperatively collected shed blood after elective cardiac surgery was processed by both salvage devices in a parallel set-up. Regarding the red blood cell concentrating performance, hematocrit and RBC recovery values indicated that the two used devices are equally effective. The proteins C3, C4, and D-dimer were reduced by at least 90% by both HemoClear and conventional centrifugal washing. The XTRA™ reached even a reduction of more than 99% with the amount of washing solution used in this set-up.

Characterized by 4.89 ± 3.1% hemolysis, the preprocessing blood quality proved to be low, not an unexpected finding, considering that this study was designed to mimic a worst-case scenario, in which the timeframe between blood collection and processing is substantial. As described by the American Association of Blood Banks standards, shed blood should only be collected for up to 6 hours and reinfused within 4 hours after processing [21, 22]. Processing in this study occurred at around 20 hours postsurgery. The rationale behind the prolonged collection time was that elevated solute concentrations would improve the detection of differences in washing performance, compared to the case when preprocessing samples contained little contaminants to be washed out.

Nevertheless, the long collection time also entails limited clinical transferability of results. The level of heparin, for example, is clinically relevant but could not be studied in this design due to this substance's half time of 60–90 minutes. Moreover, an assumption inherent to the filtration technology is that all solutes smaller than 2.3 microns should be washed out. This was the rationale for not taking measurements of a large array of solutes. However, as D-dimer, complement 3, and complement 4 loads are reduced to different extents, solubility might significantly contribute to the removal of solutes.

The HemoClear device reduced hemolysis by 4.7-fold and, showing equal performance to that of the XTRA™. The total load of free hemoglobin in the HemoClear produced concentrate (0.13 ± 0.070 versus XTRA™ 0.093 ± 0.033, *P* = 0.010) was reduced by 84.5 percent. This clearance is in line with previous reports stating free hemoglobin reductions between 53 and 99 percent [23]. The remaining free hemoglobin load may be a result of induced hemolysis [8, 24]. Various previous studies on centrifugation-based washing systems suggested that hemolysis occurs during washing due to substantial induced mechanical stress [25, 26]. Interestingly, both devices seemed to wash out free hemoglobin less effectively than the other noncellular components. This might indicate that more hemoglobin was freed during the washing due to mechanically induced hemolysis, either by the centrifugal forces of the XTRA™ or by the cross-flow of the RBCs through the pore channels of HemoClear. Another finding supporting this hypothesis is the lost percentage of RBCs that were not recuperated (HemoClear 5.2%; XTRA™ 3.5%; *P* = 0.19).

Additional research into the filtration-induced hemolysis and effects of dilution should be conducted to obtain deeper insight. Nevertheless, despite the possibly induced lysis, the total free hemoglobin load and the remaining percentage of free hemoglobin after washing are similar for HemoClear and XTRA™. These findings are also in line with previously recorded measurements for other cell salvage devices and support the washing performances of HemoClear [27].

HemoClear-processed concentrate contained a significantly higher concentration of thrombocytes (30.8 ± 21.9 10^9^/L versus XTRA™ 16.2 ± 19.0 10^9^/L, *P*=0.003) compared to the XTRA™-processed cells. However, unexpectedly, the filtrate (33.8 ± 18.8 10^9^/L, versus XTRA™ 19.8 ± 16.4 10^9^/L, *P* < 0.001) contained quite some thrombocytes too. Given that the preprocessed thrombocyte levels were far below clinical baseline, it is likely that postprocessed thrombocyte levels were below detection levels. Therefore, no conclusions regarding thrombocyte recoveries are drawn. To study thrombocyte recovery and function, future research should include simulated washing of whole blood.

Centrifugation devices have long been the cornerstone of cell salvage due to the capability to time-efficiently separate high volumes of blood cells from fluids and unwanted solutes [28]. However, although these devices enable qualitative washing, they also require substantial capital investment and often are not affordable for routine use, nor available outside of advanced operating rooms or in poor-resource settings [10, 21, 29–31].

This feasibility pilot study shows that HemoClear, a filtration-based, low-cost (anticipated pricing below 400 EUR), disposable device, can effectively wash shed blood to produce concentrated red blood cells of quality comparable to that of a centrifugation device. Moreover, HemoClear's simplistic design without mechanical requirements renders trained operators superfluous and potentially decreases the hazard of human error. Altogether, the investigated disposable HemoClear filter presents an attractive novel washing method for retained autologous RBCs, especially in settings where conventional cell savers are unavailable or not cost-effective.

## Figures and Tables

**Table 1:** Comparison of washing performance for the HemoClear and XTRA™ devices.

	Preprocessing	Postprocessing		
		XTRA™ (*n* = 18)	HemoClear (*n* = 18)	*P* value
*Concentrate*				
Hb (mmol/L)	3.71 ± 1.1	7.83 ± 1.5	8.02 ± 1.7	0.650
HCT (%)	17.9 ± 5.8	39.1 ± 9.14	40.0 ± 8.4	0.645
RBC recovery (%)		96.5 ± 4.5	94.8 ± 4.0	0.186
Free hemoglobin (mmol/L)	0.21 ± 0.14	0.093 ± 0.033	0.13 ± 0.070	**0.010**
Remaining total free Hb (%)		12.9 ± 10.9	15.5 ± 6.9	0.187
Hemolysis (%)	4.65 ± 3.3	0.73 ± 0.30	1.00 ± 0.76	0.081
MCH (pg)	2020 ± 108	1936 ± 94	1932 ± 108	0.619
MCC (%)	20.7 ± 1.1	20.1 ± 0.6	20.1 ± 0.6	0.407
MCV (fL)	97.9 ± 6.1	96.4 ± 5.8	96.4 ± 6.2	0.808
Thrombocytes (10^9^/L)	30.7 ± 19.2^*∗*^	16.2 ± 19.0	30.8 ± 21.9^*∗*^	**0.003**
Leucocytes (10^9^/L)	6.4 ± 2.8	12.8 ± 4.8	15.2 ± 6.5	**0.021**
D-dimer (mmol/L)	29.8 ± 21.6	2.6 ± 4.2	18.3 ± 29.4	**0.020**
Remaining D-dimer (%)		0.77 ± 0.55	5.9 ± 3.0	**P** < 0.001
Complement C3 (g/L)	0.63 ± 0.13	0.038 ± 0.031	0.26 ± 0.064	**P** < 0.001
Remaining complement C3 (%)		0.83 ± 0.54	6.2 ± 3.0	**P** < 0.001
Complement C4 (g/L)^*∗*^	0.12 ± 0.03	0.0072 ± 0.006	0.052 ± 0.015	**P** < 0.001
Remaining complement C4 (%)^*∗*^		0.083 ± 0.77	6.4 ± 2.9	**P** < 0.001

*Filtrate*				
Hb (mmol/L)	3.89 ± 1.4	0.094 ± 0.11	0.21 ± 0.15	0.186
HCT (%)	17.9 ± 5.8	0.16 ± 0.50	0.47 ± 0.51	0.059
Free hemoglobin (mmol/L)	0.21 ± 0.14	0.095 ± 0.069	0.20 ± 0.13	**P** < 0.001
Remaining total free Hb (%)		103 ± 18.2	217 ± 99	**P** < 0.001
Thrombocytes (10^9^/L)	30.7 ± 19.2^*∗*^	19.8 ± 16.4	33.8 ± 18.8^*∗∗*^	**P** < 0.001
Leucocytes (10^9^/L)^*∗*^	6.4 ± 2.8	0.14 ± 0.10	0.10 ± 0.00	0.120

^*∗*^ *n* = 17; ^*∗∗*^*n* = 16; below lower detection limit. *P* values in **bold** indicate statistically significant (*P* < 0.05) difference between the HemoClear and XTRA™ washing procedure.

## Data Availability

The data used to support the findings of this study are available from the corresponding author upon request.

## Abbreviations

EDTA: Ethylenediaminetetraacetic acid
Hb: Hemoglobin
HCT: Hematocrit
MCH: Mean corpuscular hemoglobin
MCV: Mean corpuscular volume
RBC: Red blood cell
XTRA™: XTRA™ autotransfusion device by LivaNova
WBC: White blood cell
WHO: World Health Organization.

## Supplementary Materials

Supplementary Materials Supplemental Video 1: the HemoClear filtration method.
